# O‐linked β‐N‐acetylglucosamine (O‐GlcNAc) modification: Emerging pathogenesis and a therapeutic target of diabetic nephropathy

**DOI:** 10.1111/dme.15436

**Published:** 2024-09-16

**Authors:** Bingxue Qi, Yang Chen, Siyang Chai, Xiaodan Lu, Li Kang

**Affiliations:** ^1^ Precision Molecular Medicine Center Jilin Province People's Hospital Changchun China; ^2^ Clinical Medicine College Changchun University of Chinese Medicine Changchun China; ^3^ Division of Cellular and Systems Medicine School of Medicine, University of Dundee Dundee UK

**Keywords:** diabetic nephropathy, glomerular mesangial cells, O‐GlcNAc modification, O‐GlcNAcylation, podocytes, renal tubular cells

## Abstract

**Aims:**

O‐Linked β‐N‐acetylglucosamine (O‐GlcNAc) modification, a unique post‐translational modification of proteins, is elevated in diabetic nephropathy. This review aims to summarize the current knowledge on the mechanisms by which O‐GlcNAcylation of proteins contributes to the pathogenesis and progression of diabetic nephropathy, as well as the therapeutic potential of targeting O‐GlcNAc modification for its treatment.

**Methods:**

Current evidence in the literature was reviewed and synthesized in a narrative review.

**Results:**

Hyperglycemia increases glucose flux into the hexosamine biosynthesis pathway, which activates glucosamino‐fructose aminotransferase expression and activity, leading to the production of O‐GlcNAcylation substrate UDP‐GlcNAc and an increase in protein O‐GlcNAcylation in kidney cells. Protein O‐GlcNAcylation regulates the function of kidney cells including mesangial cells, podocytes, and proximal tubular cells, and promotes renal interstitial fibrosis, resulting in kidney damage. Current treatments for diabetic nephropathy, such as sodium‐glucose cotransporter 2 (SGLT‐2) inhibitors and renin–angiotensin–aldosterone system (RAAS) inhibitors, delay disease progression, and suppress protein O‐GlcNAcylation.

**Conclusions:**

Increased protein O‐GlcNAcylation mediates renal cell damage and promotes renal interstitial fibrosis, leading to diabetic nephropathy. Although the full significance of inhibition of O‐GlcNAcylation is not yet understood, it may represent a novel target for treating diabetic nephropathy.

## Graphical abstract

O‐Linked β‐N‐acetylglucosamine (O‐GlcNAc) modification is increased in diabetic nephropathy and contributes to its pathogenesis and progression. Current treatments of diabetic nephropathy, including sodium‐glucose cotransporter 2 (SGLT‐2) inhibitors and renin‐angiotensin‐aldosterone system (RAAS) inhibitors, delay the progression of diabetic nephropathy and suppress protein O‐GlcNAcylation. Although the full significance of inhibition of O‐GlcNAcylation is not yet understood, it may represent a novel target for the treatment of diabetic nephropathy.

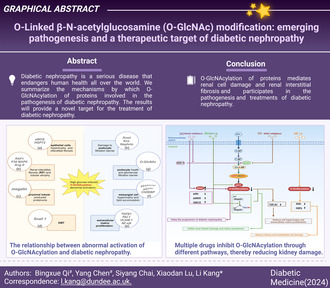

**What's New?**

- Abnormal activation of O‐linked β‐N‐acetylglucosamine (O‐GlcNAc) modification is implicated in diabetic nephropathy.
- This review article summarizes the mechanisms by which O‐GlcNAcylation of proteins mediates renal cell damage and promotes renal interstitial fibrosis, leading to diabetic nephropathy. Current treatments of diabetic nephropathy have also been shown to regulate protein O‐GlcNAcylation.
- O‐GlcNAcylated proteins may become valuable markers of diabetic nephropathy and represent as new targets for future treatments of the disease.

## 1 OVERVIEW OF O‐LINKED Β‐N‐ACETYLGLUCOSAMINE (O‐GlcNAc) MODIFICATION IN DIABETIC NEPHROPATHY

The prevalence of diabetes is increasing rapidly and has become a serious public health challenge worldwide. The latest global data show that 529 million people in the world population are living with diabetes in 2021, and more than 1.31 billion people are projected to have diabetes by 2050.^1^ Diabetic nephropathy is a microvascular complication of diabetes and has become the leading cause of chronic kidney disease (CKD) and end‐stage renal disease (ESRD) in most developed countries.^2^ In the United States, up to 44% of ESRD cases are linked to diabetes.^3^ Diabetic nephropathy substantially elevates the risk of cardiovascular disease and related morbidity and mortality.^4^ Furthermore, patients with diabetic nephropathy may require maintenance dialysis or renal transplantation, and these treatments impose considerable financial and psychological burdens.^5^ According to statistics, one in three adults with diabetes has diabetic nephropathy, but most people are not aware. Therefore, increasing awareness of diabetic nephropathy is crucial for early intervention, alleviating the economic burden and reducing complications and mortality.

The first‐line treatment of diabetic nephropathy is to treat and control diabetes (e.g. insulin, metformin, glucagon‐like peptide 1 receptor agonists (GLP‐1RA), and glucose co‐transporter 2 inhibitors (SGLT‐2i)) and high blood pressure (e.g., angiotensin‐converting enzyme (ACE) inhibitors and angiotensin receptor blockers (ARBs)). Finerenone, a novel mineralocorticoid receptor antagonist (MRA) medication, is used to reduce tissue scarring in diabetic nephropathy and research shows that it decreases the risk of kidney failure and death related to cardiovascular events in adults with Type 2 diabetes, via its anti‐inflammatory and anti‐fibrotic action.^6^ The treatment of more advanced diabetic nephropathy (ESRD or kidney failure) often involves invasive hemodialysis and kidney transplant. Despite the current treatment strategies for diabetic nephropathy, no treatment works directly in the kidney to repair, reverse or delay kidney damage. The development of future treatments of diabetic nephropathy will require better understanding of its pathogenesis.

Several mechanisms are involved in the pathogenesis of diabetic nephropathy, including the epithelial‐to‐mesenchymal transition (EMT) within the renal tubules,^7^ extensive accumulation of the extracellular matrix,^8^ increased production of reactive oxygen species (ROS) and oxidative stress,^9^ inflammation,^10^ decreased autophagy,^11^ dysregulated cell proliferation,^12^ mitochondrial dysfunction,^13^ and accumulation of advanced glycation end products (AGEs).^14^ In recent years, abnormal activation of O‐GlcNAc modification has been implicated in diabetic nephropathy.^15^, ^16^, ^17^

O‐GlcNAc modification, discovered by G.W. Hart et al in 1984, is different from the traditional glycosylation and has important biological functions.^18^ O‐GlcNAc modification is a unique type of post‐translational modification of proteins, defined by adding or removing a single monosaccharide β‐O‐d‐N‐acetylglucosamine (β‐O‐GlcNAc) to the serine or threonine residue of proteins by O‐GlcNAc transferase (OGT) and O‐GlcNAcase (OGA) without modifying the tyrosine residues. O‐GlcNAc modification sites are often the same or adjacent to the phosphorylation sites, enabling direct or indirect interactions between O‐GlcNAcylation and phosphorylation.^19^, ^20^ O‐GlcNAcylation can sense environmental changes, regulate protein–protein interactions, and participate in many important biological processes such as gene expression, signal transduction, nucleocytoplasmic dynamics,^21^ and cell cycle and DNA damage.^22^ O‐GlcNAcylation promotes proliferation and activation of T and B cells, regulates inflammatory and antiviral responses of macrophages, promotes activation of neutrophils, and inhibits the activity of nature killer cells.^23^

In the kidney, the involvement of O‐GlcNAc modification in the pathogenesis of diabetic nephropathy has only recently emerged. Increased O‐GlcNAcylation disrupts the function and metabolic balance of kidney cells, resulting in excessive extracellular matrix accumulation and thickening of the basement membrane. These further cause pathological changes in the renal structure and impair renal filtration barrier, leading to proteinuria and renal dysfunction.^24^, ^25^, ^26^, ^27^ In diabetic nephropathy, O‐GlcNAcylation also contributes to renal fibrosis.^28^, ^29^

O‐GlcNAc modification has also been implicated in the current treatments of diabetic nephropathy. The renin–angiotensin–aldosterone system (RAAS) inhibitors prevent elevation of protein O‐GlcNAcylation in diabetic kidney.^15^ SGLT‐2i dapagliflozin decreases O‐GlcNAcylation and tubular hypoxia, ameliorating renal fibrosis.^30^ Moreover, SGLT‐2i improves megalin endocytic function through suppressing its O‐GlcNAcylation and protects the diabetic kidney from protein overload.^31^ These evidences provide insights for targeting O‐GlcNAcylation for novel therapies for the treatment of diabetic nephropathy.

## 2 MOLECULAR REGULATION OF O‐GlcNAc MODIFICATION

**FIGURE 1:**
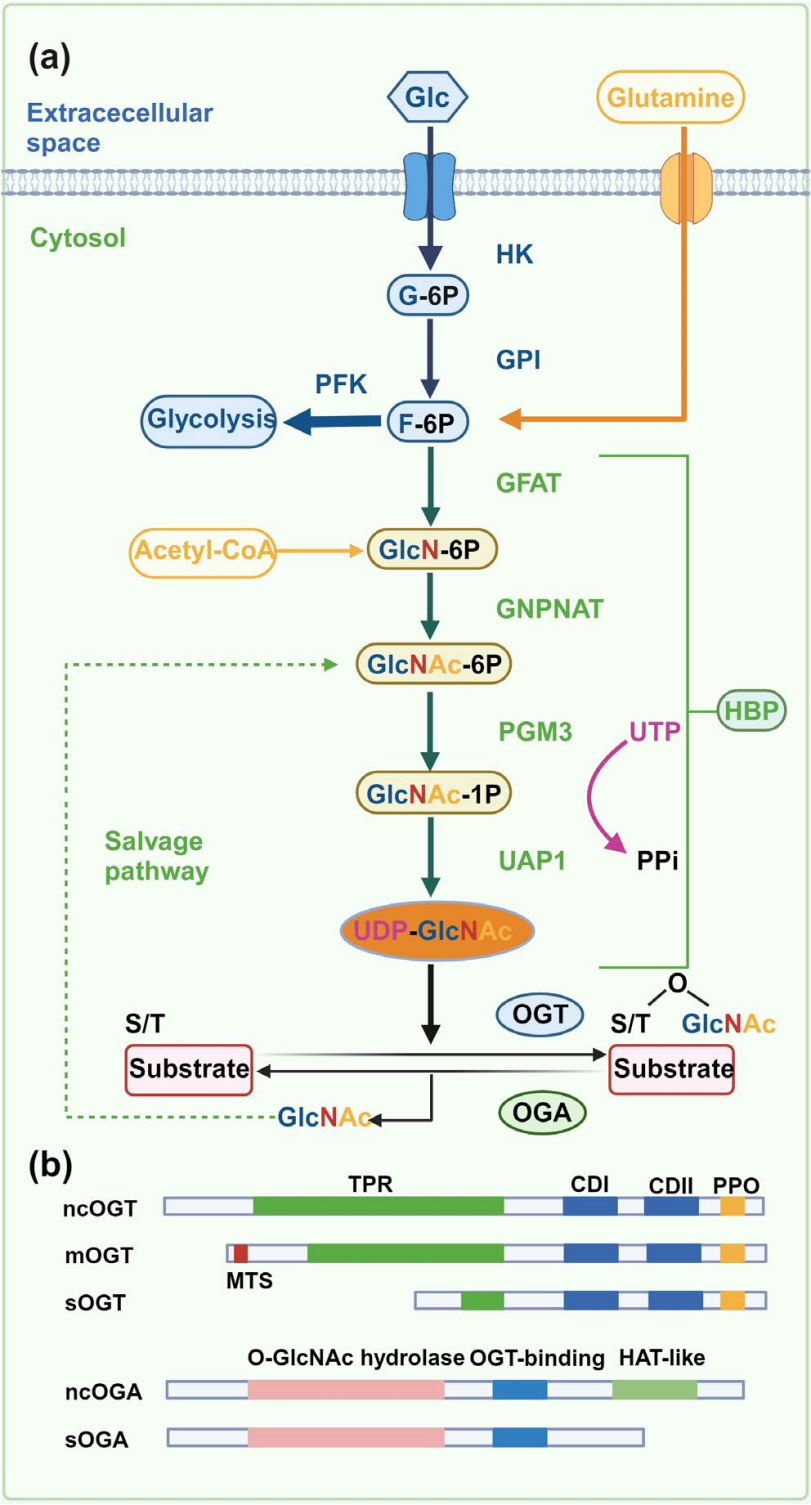
Overview of the hexosamine biosynthetic pathway (HBP) and O‐GlcNAcylation. (a) HBP is a small branch of glycolysis where glucose (Glc) becomes fructose‐6‐phosphate (F‐6P) through the first two steps shared by HBP and glycolysis pathways. Only 2%–3% of F‐6P enters HBP. After glucose enters the cell, glucose is rapidly phosphorylated to glucose‐6‐phosphate (glucose‐6P) by hexokinase (HK). Glucose‐6p is isomerized by phosphoglucose isomerase (PGI) to produce F‐6P, a substrate for phosphofructokinase (PFK) or glucosamino‐fructose aminotransferase (GFAT) of the glycolytic pathway, which is a rate‐limiting reaction of the HBP. GFAT requires glutamine (Gln) as an amine donor to produce glucosamine 6‐phosphate (GlcN‐6P), which is then n‐acetylated by glucosamine 6‐phosphate n‐acetyltransferase (GNA1) to produce n‐acetyl‐glucosamine 6‐phosphate (GLCN‐6P). This step requires acetyl‐CoA as an acetyl donor. Glnac‐6p is converted to GLNAC‐1P by acetylglucosamine phosphate mutase (PGM‐3). Using UTP as a nucleotide donor, UDP‐N‐acetylglucosamine pyrophosphorylase (UAP1) generates UDP‐GlcNAc.^139^ (b) O‐GlcNAc transferase (OGT) and O‐GlcNAcase (OGA) catalyze the addition and removal of O‐GlcNAc, respectively. Free GlcNAc can be recovered by the GlcNAc rescue pathway. This pathway converts GlcNAc to GlcNAc‐6‐phosphate (GlcNAc‐6P), which can be utilized by HBP. OGT and OGA isomers schematic. The nucleocytoplasmic (ncOGT), mitochondrial (mOGT), and short (sOGT) isoforms of OGT differ in length due to the number of amino‐terminal (N‐terminal) tetrapeptide repeat sequences (TPRs). However, they share common carboxy‐terminal (C‐terminal) catalytic (CDI and II) and phospholipid‐binding (PPO) structural domains. The unique N‐terminal mitochondrial targeting sequence (MTS) is present in mOGT. The ncOGA and sOGA isoforms of OGA share the same N‐terminal O‐GlcNAc hydrolase structural domain and the central OGT binding region; however, sOGA lacks the C‐terminal histone acetyltransferase‐like (HAT‐like) structural domain found in ncOGA.

Protein O‐GlcNAcylation occurs in the nucleus, cytoplasm, and mitochondrion.^19^, ^32^, ^33^ O‐GlcNAcylation, as a nutrient sensor is involved in the regulation of a wide range of cellular events, including epigenetics, transcription, signalling, and rhythm.^18^, ^32^, ^33^, ^34^, ^35^ O‐GlcNAc modification also cross talks with phosphorylation by modifying the same or proximal sites of target proteins. Unlike conventional glycosylation, O‐GlcNAc modifications do not usually elongate or produce complex branched glycan structures. Instead, they act through the two highly conserved enzymes in eukaryotes: OGT and OGA. OGT catalyzes the addition of β‐O‐GlcNAc utilizing the donor substrate UDP‐GlcNAc, and OGA catalyzes the removal of O‐GlcNAc and inhibits OGT activity.^36^, ^37^ UDP‐GlcNAc is synthesized from glucose via the hexosamine biosynthesis pathway (HBP) (Figure 1a). Because UDP‐GlcNAc is involved in nucleotide, carbohydrate, fatty acid, and amino acid metabolism, it is considered as a central metabolic sensor.^38^, ^39^

Human OGT encodes three isoforms, all of which contain a C‐terminal catalytic domain but differ in the number and subcellular localisation of N‐terminal tetrapeptide repeats (TPRs) due to selective splicing of the transcript in the N‐terminal region, classifying them as nucleocytoplasmic OGT (ncOGT), mitochondrial OGT (mOGT), and short OGT (sOGT) (Figure 1b). ncOGT contains 13 TPRs with a molecular weight of 116 kDa, whereas mOGT has a molecular weight of 103 kDa, with only 9 TPRs and a selectively spliced N‐terminal mitochondrial targeting sequence (Figure 1b). sOGT has a molecular weight of 78 kDa and contains 2 TPRs.^40^ ncOGT and sOGT are located in the nucleus and cytoplasm; however, mOGT tends to accumulate in the inner mitochondrial membrane.^41^ Substrate selection of OGT is mainly regulated by ncOGT, thus a major participant in intracellular O‐GlcNAc modification. The N‐terminal TPR region of OGT regulates a variety of cellular processes, including cell cycle,^42^, ^43^, ^44^ transcriptional regulation,^45^, ^46^, ^47^ and protein transport.^48^ It regulates interactions between target and regulatory proteins.^49^, ^50^, ^51^ The N‐terminal TPR region also facilitates the multimerization of OGT and acts as a docking site for protein substrate recognition and glycoside selection.

OGA is highly conserved across species and is essential for mammals and plants.^52^ It is widely expressed in tissues including pancreas, brain, and skeletal muscle.^53^, ^54^, ^55^ OGA isoforms, the nucleocytoplasmic OGA (ncOGA), and the short OGA (sOGA) are encoded by two major splice variants that differ in the presence or absence of a C‐terminal region, respectively (Figure 1b). ncOGA consists of a catalytic domain at the N‐terminus, similar to that of the CAZy‐glycosyl hydrolase family 84 (GH84), and a pseudohistone acetyltransferase (HAT) domain at the C‐terminus, linked by an ordered stem structural domain. The molecular weight of full‐length ncOGA is 130 kDa. OGA mRNA undergoes selective splicing in the HAT structural domain,^56^, ^57^ resulting in sOGA. sOGA is 100 kDa. Like OGT, OGA can bind to multiple protein substrates.

## 3 MOLECULAR MECHANISMS OF ABNORMAL O‐GlcNAc MODIFICATION IN THE PATHOGENESIS OF DIABETIC NEPHROPATHY

### 3.1 O‐GlcNAc modification regulates mesangial cell function

Mesangial cells originate from the posterior renal interstitium, which participates in the construction of glomerular microvascular bed and the production of mesangial matrix for the maintenance of glomerular homeostasis. Clinical studies have shown that lipid accumulation and lipid metabolism abnormalities in glomeruli lead to lipotoxicity in the kidney, accelerating the progression of diabetic nephropathy.^58^, ^59^

**FIGURE 2:**
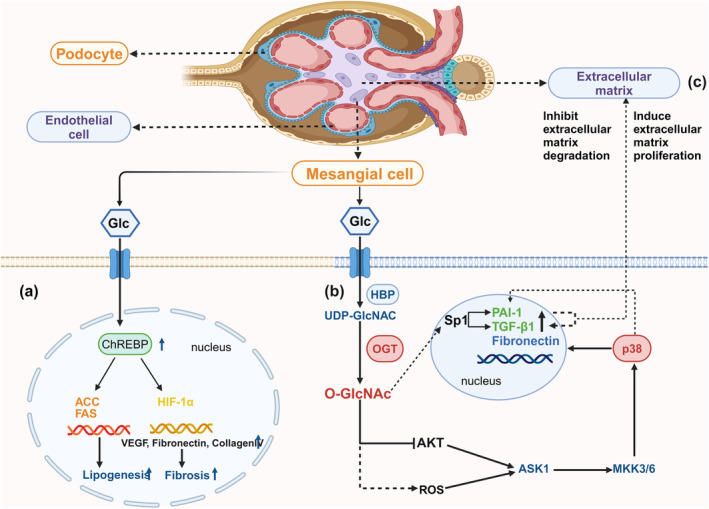
O‐GlcNAcylation is involved in mesangial cell fibrosis and extracellular matrix accumulation, damaging renal function. (a) In mesangial cells, high glucose induces O‐GlcNAcylation of ChREBP, this process triggers lipogenic enzymes and fibrosis‐related proteins, leading to lipid accumulation in mesangial cells and accelerated kidney damage. (b) O‐GlcNAcylation promotes the activation of ASK1 and p38 MAPK pathways by inhibiting Akt phosphorylation in mesangial cells. Upregulated p38 induces the expression of PAl‐1, fibronectin, and TGF‐β1, leading to diabetic glomerulosclerosis. In addition, O‐GlcNAcylation may also stimulate ASK1 by increasing ROS. (c) Under high glucose conditions, O‐GlcNAcylation increases, promotes the expression of Sp1, induces the production of PAI‐1 and TGF‐β1, inhibits the degradation of extracellular matrix, and promotes the occurrence and development of CKD. ACC, Acetyl‐CoA carboxylase; ASK1, Apoptosis signal‐regulating kinase 1; FAS, Fatty acid synthase; Glc, Glucose; HIF‐1α, Hypoxia‐inducible factor 1‐α; MKK3/6, MAPK kinases; VEGF, Vascular endothelial growth factor.

High glucose increases O‐GlcNAc modification of carbohydrate response element binding protein (ChREBP) in mesangial cells (Figure 2a). ChREBP is a basic helix–loop–helix leucine zipper transcription factor, a key determinant of lipid synthesis by regulating the expression of lipogenic genes containing carbohydrate response element (ChRE).^60^ Modification of ChREBP by O‐GlcNAc glycosylation can regulate its transcriptional activity, stability, and/or subcellular localisation.^61^ O‐GlcNAc modification is crucial for the glucose response of ChREBP as it is shown that high glucose‐induced O‐GlcNAcylation of ChREBP promotes lipid accumulation in mesangial cells.^62^ Notably, ChREBP O‐GlcNAcylation also increases fibrosis‐related gene expression in mesangial cells under high glucose.^62^

Mechanistically, increased O‐GlcNAcylation in mesangial cells under high glucose promotes fibrotic signalling by the activation and phosphorylation of p38 mitogen‐activated protein kinase (MAPK) and Jun N‐terminal kinase (JNK) (Figure 2b).^24^, ^25^ While a reduction of O‐GlcNAcylation by inhibiting OGT decreases phosphorylation of p38 MAPK and JNK, an increase in O‐GlcNAcylation by inhibiting OGA increases p38 MAPK phosphorylation.^25^ Activated p38 MAPK leads to increased expression of transforming growth factor β1 (TGF‐β1), plasminogen activator inhibitor‐l (PAI‐1), and fibronectin, contributing to excessive matrix accumulation seen in diabetic nephropathy.^25^ Therefore, O‐GlcNAcylation in mesangial cells mediates kidney injury by promoting activation of profibrotic p38 MAPK in response to high glucose. Furthermore, increased O‐GlcNAcylation causes mesangial microvascular damage through regulating specific protein 1 (Sp1) transactivation in mesangial cells.^24^

Mesangial cell hypertrophy and increased secretion of matrix proteins lead to glomerular enlargement, which is thought to be one of the earliest alterations in diabetic nephropathy. Mesangial cells in the diabetic state exhibit transient and limited proliferation in the early stages of growth, followed by growth arrest and hypertrophy.^26^ Masson et al. treated mesangial cells with glucosamine (GlcN) to activate HBP and found that GlcN arrested cells in the G0/G1 phase by increasing the expression of cyclin‐dependent kinase inhibitor p21^Waf1/Cip1^, thereby inducing mesangial cell hypertrophy.^26^ In addition, in an animal model of streptozotocin (STZ)‐induced diabetes, a model of early stage of diabetic nephropathy, mesangial cells are arrested in the G1 phase and hypertrophic, and extracellular matrix protein synthesis and deposition are increased.^63^ Mesangial cells of STZ‐induced diabetic rats and mice also showed increased expression of p21^Waf1/Cip1^ and cyclin‐dependent kinase inhibitor p27^KIP1^.^64^, ^65^ During the cell cycle, the levels of OGT, OGA, and O‐GlcNAc fluctuate significantly, therefore O‐GlcNAcylation and alterations of HBP flux are emerging as important regulators of cell cycle progression.^66^ High glucose increases HBP flux, enhances the production of ROS (Figure 2b), and causes cellular oxidative stress, extracellular matrix deposition, and mesangial cell hypertrophy.^67^, ^68^, ^69^, ^70^, ^71^

### 3.2 O‐GlcNAc modification regulates podocyte function

**FIGURE 3:**
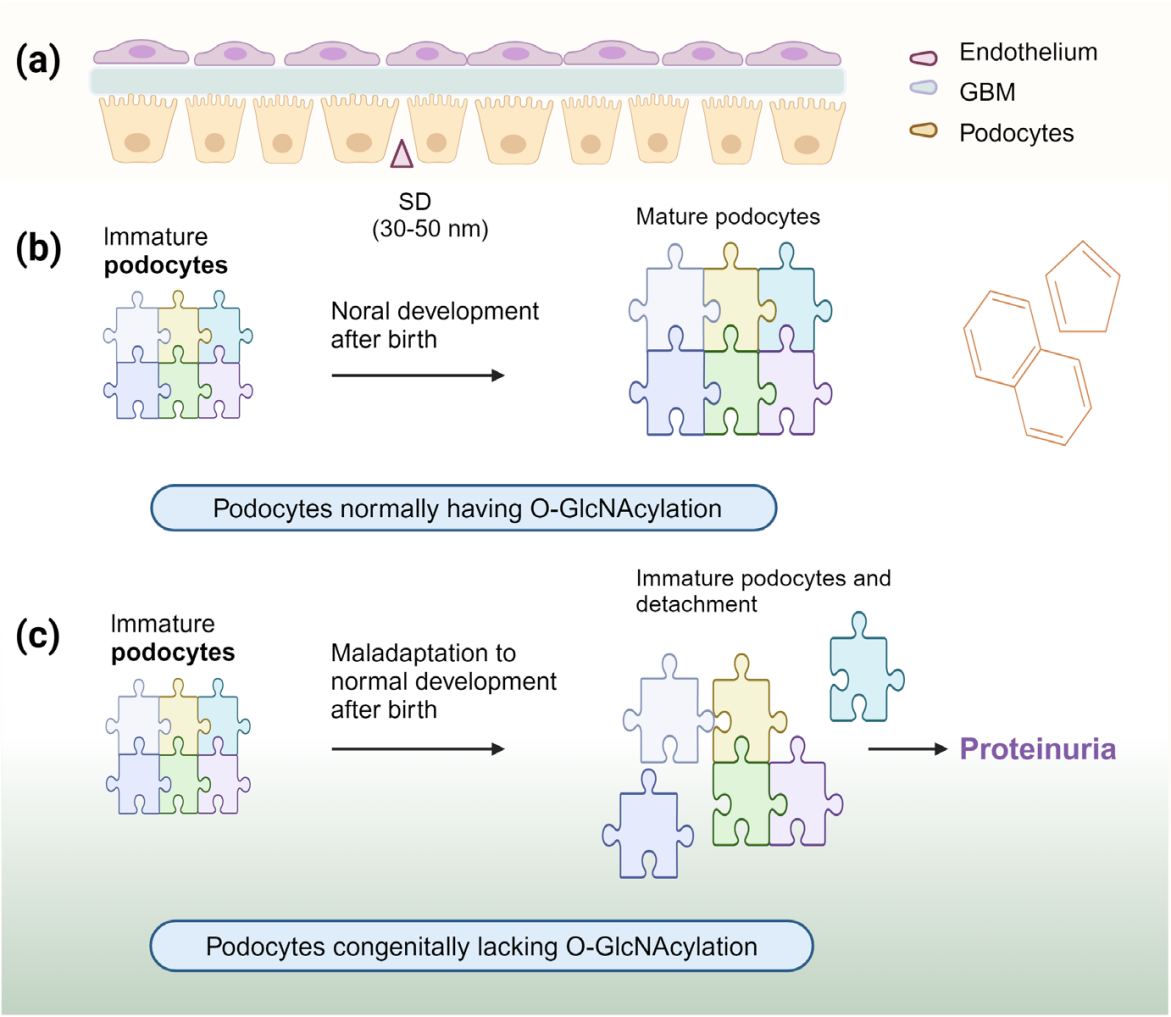
O‐GlcNAcylation is involved in mediating the normal physiological structure and function of podocytes. (a) Glomerular podocytes, endothelial cells and the glomerular basement membrane (GBM) together form the glomerular filtration barrier (GFB).^140^ Among them, podocytes are the last barrier to GFB. (b) After birth, glomerular size increases with normal body growth, but the number of podocytes does not. Therefore, normal podocytes require hypertrophy and/or foot process elongation to maintain glomerular filtration. (c) Podocyte loss as well as proteinuria is observed in mice with congenital lack of O‐GlcNAcylation in podocytes.

Podocyte loss is an early event of diabetic nephropathy.^72^ Hyperglycemia triggers podocytes to undergo morphological changes, cell shedding, and cell apoptosis, among others.^73^ Podocytes cover the outside of the glomerular basement membrane (GBM) and form the ultimate barrier against protein loss (Figures 2c and 3a). It has been demonstrated that podocyte injury leads to proteinuria, followed by glomerular sclerosis contributing to impaired glomerular filtration, as the decrease in podocyte numbers in humans with diabetes is proportional to the severity of the injury and the degree of proteinuria.^72^, ^74^, ^75^, ^76^, ^77^, ^78^ Growing evidence from experimental models show that podocyte loss and dysfunction are strongly associated with glomerulosclerosis.

Podocytes consist of a cell body with numerous primary and secondary foot processes. Podocytes and their foot processes line the outside of the capillaries and mesangial cells, preventing the leakage of proteins and other macromolecules from the blood into the urine.^79^, ^80^ Studies have shown the presence of O‐GlcNAcylated proteins in the cytoplasm and nucleus of podocytes.^27^ OGT‐mediated O‐GlcNAcylation is essential for complete maturations of mouse podocytes.^27^ Podocyte‐specific OGT knockout mice develop glomerulosclerosis, tubulointerstitial lesions, impairment in podocyte foot processes, and decreased podocyte protein expression during growth (Figure 3b,c).^27^ Cytoskeletal proteins, such as β‐actin, α‐actin 4, and α‐integrins, are important in maintaining the structure and movement of podocyte foot processes, allowing adaptive volumetric responses to changes in the environmental tone.^16^, ^81^ Studies have found that O‐GlcNAcylation of β‐actin in podocytes of the glomeruli contributes to the morphological changes in the glomeruli and tubules of non‐obese diabetic rats as well as human diabetic kidneys.^82^

### 3.3 O‐GlcNAc modification regulates renal tubular function

Renal tissue scarring or renal interstitial fibrosis (RIF) is a pathological and dynamic process manifested in diabetic nephropathy. It includes increased matrix production, inhibition of matrix degradation, increased cell–matrix interactions, activation of mesangial cells and myofibroblasts, tubular EMT or partial EMT, and immune cell infiltration.^83^ Many studies have attempted to delay the progression of diabetic nephropathy by inhibiting RIF, among which EMT has been found to be one of the most critical mechanisms of RIF.^84^, ^85^

**FIGURE 4:**
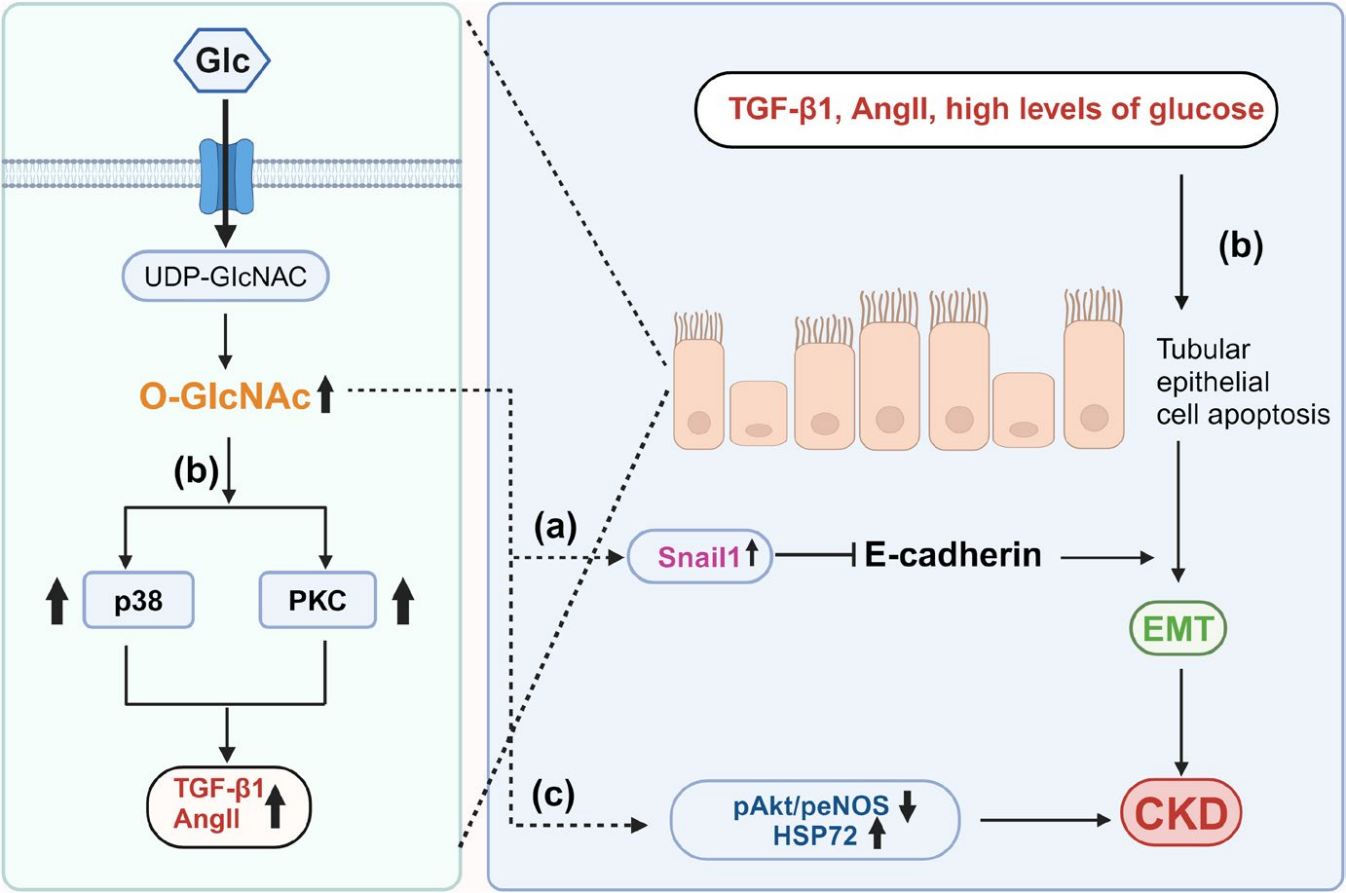
O‐GlcNAcylation is involved in regulating the structure and function of renal tubular cells, promoting renal interstitial fibrosis, and accelerating the progression of renal diseases. (a) Under high glucose (Glc) conditions, the modification of O‐GlcNAc in renal cells increases, improving the stability of Snail1, and its inhibitory effect is enhanced, thereby weakening the expression of E‐cadherin mRNA, promoting EMT, and accelerates the occurrence and development of CKD. (b) In renal tubular epithelial cells under a high glucose environment, O‐GlcNAcylation increases, inducing the expression of p38 and PKC, promoting the production of TGF‐β1 and Ang II, causing renal tubular cell apoptosis and EMT, leading to renal tubular atrophy. (c) O‐GlcNAcylation increases in renal tubular epithelial cells under high glucose conditions, peNOS and pAkt levels decreased, while HSP72 levels increased and promotes the progression of CKD.

O‐GlcNAcylation is involved in the regulation of EMT during RIF. It was found that O‐GlcNAcylation‐induced EMT in human proximal tubular HK2 cells.^28^ In addition, mass spectrometry studies have identified that RAF1 is a potential substrate of O‐GlcNAcylation.^28^ O‐GlcNAcylation of RAF1 increases its stability by inhibiting ubiquitination. Importantly, the study showed that O‐GlcNAcylation of RAF1 were upregulated in the kidney tissue of unilateral ureteral obstructioin rats, a model of renal fibrosis and tubular injury.^28^ Furthermore, Snail1 (zinc finger protein transcription factor l) is an important regulator of EMT. High glucose increases O‐GlcNAcylation but decreases phosphorylation of Snail1 in human embryonic kidney cells, suppressing the transcription of E‐cadherin and inducing EMT (Figure 4a).^86^

In proximal tubular epithelial cells (PTECs), high glucose promotes an increase in HBP glucose flux, increases protein O‐GlcNAcylation, activates RAAS and causes PTEC hypertrophy, ultimately leading to RIF (Figure 4b).^87^ Furthermore, PTECs cultured under high glucose or isolated from diabetic rats have increased O‐GlcNAcylation and decreased phosphorylation of endothelial nitric oxide synthase (eNOS) and heat shock protein (HSP) 72, inducing RIF (Figure 4c).^15^ Other studies showed that O‐GlcNAcylation suppressed phosphorylation of eNOS and protein kinase B or Akt16, which increased expression of α‐actinin and promoted structural changes in PTEC microvilli.^88^, ^89^ Apart from these, high levels of O‐GlcNAcylation can also increase blood pressure, resulting in downregulation of megalin, a receptor that mediates endocytosis in PTECs.^90^ High glucose‐induced O‐GlcNAcylation leads to proteinuria by decreasing the activity of protein kinase B, and thereby decreasing expression of megalin and subsequently decreasing reabsorption of albumin in the proximal tubules.^91^

In addition, lipolysis is important in maintaining energy homeostasis in PTECs, and lipid metabolism is dysregulated in patients with diabetic nephropathy.^92^ Genetic deletion of OGT in mice causes Fanconi syndrome‐like abnormalities, PTEC apoptosis, reduced PETC lipolysis and ATP production, severe tubular cell damage, and enhanced lipotoxicity after fasting for 48 hours.^92^ This study suggest that O‐GlcNAc modification is critical for normal renal energy homeostasis as well as PTEC function and survival. These results are consistent with the pro‐survival role of O‐GlcNAcylation,^93^ as increased O‐GlcNAcylation levels promote the translocation of HSPs and the expression of HSP72, one of the important regulators of cell protection in the kidney.^94^ Although increased protein O‐GlcNAcylation are often considered pathogenic, the studies by Sugahara et al. and others suggest that O‐GlcNAcylation defects could also cause renal tubular damages during fasting and diabetes.^92^

### 3.4 O‐GlcNAc modification regulates extracellular matrix proliferation

TGF‐β1 promotes the production of extracellular matrix proteins, and its overexpression leads to extracellular matrix proliferation.^95^ Under high glucose, HBP flux increases, thus increasing O‐glycosylation activity, which increases the nuclear level of upstream stimulatory factor (USF) and its binding with ChRE in glomerular mesangial cells and proximal tubule cells.^96^, ^97^, ^98^ Nuclear expression of USF induces TGF‐β1 promoter activity, promoting extracellular matrix proliferation in diabetic nephropathy.^96^, ^97^, ^98^

Upon O‐GlcNAcylation, Sp1 binds to the promoter region of PAI‐1, inducing gene expression of PAI‐1 and TGF‐β1 (Figure 2c).^24^, ^99^ PAI‐1 inhibits the degradation of extracellular matrix, leading to progressive stacking of matrix proteins in diabetic nephropathy. In parallel, O‐GlcNAc depletion prevents high‐glucose‐induced phosphorylation of p38 MAPK and JNK in rat mesangial cells and thus decreases the expression of PAI‐1 and TGF‐β1, reducing extracellular matrix deposition.^25^

Inflammatory cell infiltration contributes to the development and progression of diabetic nephropathy through promoting extracellular matrix proliferation..^100^ Chemokines and adhesion molecules are key mediators of renal injury by attracting circulating white blood cells and facilitating transmigration of these cells into renal tissues. Biopsy specimens of diabetic kidneys have increased expression of adhesion molecules such as vascular cell adhesion molecule‐1 (VCAM‐1) and intercellular adhesion molecule‐1 (ICAM‐1).^101^ In glomerular mesangial cells, high glucose increases glucosamino‐fructose aminotransferase (GFAT) expression and O‐glycosylation of p65/Rel, a nuclear factor kappa B (NF‐κB) subunit, which promote VCAM‐1 expression by activating NF‐κB. Inactivation of the two NF‐κB binding sites in the VCAM‐1 promoter abolishes VCAM‐1 transcription in response to high glucose, glucosamine, and GFAT overexpression.^100^ In addition, O‐GlcNAcylation inhibits the activity of inducible nitric oxide synthase (iNOS), reduces nitric oxide production, and enhances the expression of pro‐inflammatory cytokines such as IL‐6, IL‐1β, and IL‐12.^102^ O‐GlcNAcylation can also directly activate NF‐κB and its downstream effectors, enhancing pro‐inflammatory cytokine signaling and activating other innate immune cell function to promote inflammation, which is positively correlated with the expression of OGT.^103^, ^104^, ^105^, ^106^, ^107^

### 3.5 O‐GlcNAc modification regulates insulin sensitivity and insulin secretion outside the kidney

Outside the kidney, O‐GlcNAc modification can indirectly contribute to the progression of diabetic nephropathy through regulating insulin sensitivity and insulin secretion. Increased O‐GlcNAcylation level is associated with insulin resistance in multiple tissues, such as adipose tissue and skeletal muscle.^108^, ^109^ When 3T3‐L1 preadipocytes were treated with glucosamine and OGA inhibitor PUGNAc (1,5‐hydroximolactone) to stimulate O‐GlcNAcylation of total proteins, O‐GlcNAcylation level positively correlated with insulin resistance and endoplasmic reticulum stress of the cells.^110^ Moreover, suppression of O‐GlcNAcylation of total proteins by the shRNA‐mediated silencing of OGT, improved insulin sensitivity and alleviated endoplasmic reticulum stress in insulin‐resistant preadipocytes.^110^ O‐GlcNAc modification has the potential to impair insulin receptor signaling as insulin receptor substate‐1 (IRS‐1) (Ser1101) and IRS‐2 (Ser1149) become glycosylated following an increase in UDP‐GlcNAc pools, contributing to insulin resistance.^111^

Increased protein O‐GlcNAcylation is also associated with hyperglycemia‐induced glucose toxicity, β cell apoptosis, and impaired insulin secretion.^112^ STZ or glucosamine‐induced elevation of nucleocytoplasmic protein O‐GlcNAcylation was accompanied by impaired insulin secretion and enhanced apoptosis of pancreatic β cells.^112^ Moreover, β cell‐specific OGT‐deficient mice displayed hyperglycemia with insulin depletion accompanied by β cell apoptosis.^113^

## 4 O‐GlcNAcylation AS A THERAPEUTIC TARGET FOR DIABETIC NEPHROPATHY

### 4.1 GFAT inhibitors

**FIGURE 5:**
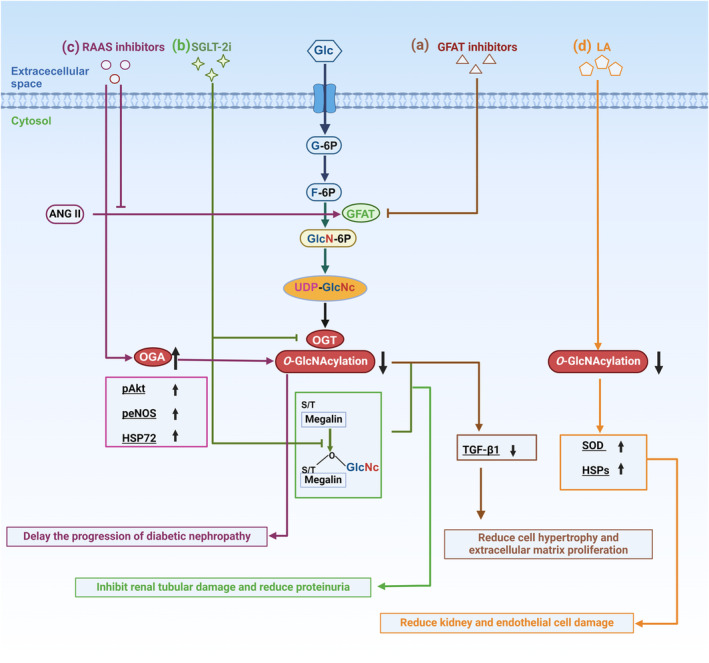
Multiple drugs inhibit O‐GlcNAcylation through different pathways, thereby reducing kidney damage. (a) GFAT inhibitors inhibit GFAT enzyme activity, block glucose from entering the HBP pathway, reduce O‐GlcNAc glycosylation and TGF‐β1 levels, inhibit cell hypertrophy and extracellular matrix proliferation, and protect the kidneys. (b) SGLT‐2i inhibits SGLT2 and blocks glucose from entering the proximal tubule cells, thereby reducing HBP flux, attenuating OGT and O‐GlcNAcylation levels. SGLT‐2i also inhibits O‐GlcNAcylation of megalin, reduces the endocytosis of megalin, and improves renal function. (c) RAAS inhibitors successfully improve diabetes‐induced renal O‐GlcNAcylation mainly by increasing OGA, eNOS phosphorylation. In addition, RASS inhibitors block ANG II from activating GFAT and reduced O‐GlcNAcylation in cells. (d) LA reduces the level of O‐GlcNAcylation, upregulates the expression of SOD, and enhances the expression of HSPs. Ang II, angiotensin II; HSP, heat shock protein; GFAT, Glucosamino‐fructose aminotransferase; Glc, Glucose; LA, lipoic acid; peNOS, phosphorylated endothelial nitric oxide synthase; RAAS, Rctivates the renin‐angiotensin‐aldosterone system; SGLT‐2i, sodium‐glucose cotransporter‐2 inhibitors; SOD, CuZn‐superoxide dismutase.

GFAT is the rate‐limiting enzyme for glucose entry into the HBP pathway to produce the O‐GlcNAcylation substrate UDP‐GlcNAc.^114^ Upregulation of GFAT allows more glucose to enter the HBP pathway, thereby increasing O‐GlcNAcylation. Kidney sections from patients with Type 1 diabetes show increased GFAT expression^115^ and GFAT activity is elevated in skeletal muscle of patients with Type 2 diabetes.^116^ Moreover, RNAseq analysis of kidney biopsies from patients with diabetic nephropathy reveals an increase in the expression of GFAT.^117^ Incubation of LLC‐PK1 cells, a model of PTECs, with 6‐diazo‐5‐oxo‐L‐norleucine (DON), an inhibitor of GFAT, decreases O‐GlcNAcylation (Figure 5a).^117^ In rat primary mesangial cells, overexpression of GFAT activates PAI‐1 promoter and increases mRNA levels of TGF‐β1 and TGF‐β type I and type II receptors, which are abrogated by GFAT inhibitors *o*‐diazoacetyly‐l‐serine and 6‐diazo‐5‐oxonorleucine (Figure 5a).^118^ Furthermore, in human mesangial cells, overexpression of GFAT promotes TGF‐β1 expression via a protein kinase C and p38 MAPK‐dependent mechanism, whereas the treatment with GFAT inhibitor azaserine prevents the induction of TGF‐β1 in GFAT‐overexpressing cells (Figure 5a).^119^ Interestingly, some herbs and foods exert effects of reducing GFAT activity, therefore representing therapeutic potential. In MCGT1 cells (a GLUT1 transgenic rat mesangial cell line), rhein (an anthraquinone compound isolated from rhubarb) reduces cellular hypertrophy and extracellular matrix proliferation by inhibiting GFAT activity, decreasing the level of UDP‐GlcNAc, and thereby inhibiting O‐GlcNAc modification.^120^

### 4.2 SGLT‐2i

SGLT‐2 is expressed in the early proximal tubule of the kidney. SGLT‐2i are novel hypoglycemic agents that block SGLT‐2, leading to an increase in urinary glucose excretion and a decrease in circulating glucose concentrations.^121^, ^122^ Several studies have shown that SGLT‐2i have beneficial effects on delaying the progression of diabetic nephropathy.^123^ The use of SGLT‐2i is associated with a lower risk for dialysis and acute kidney injury in patients with Type 2 diabetes.^124^ Mechanistically, SGLT‐2i dapagliflozin treatment attenuates renal injury by reducing renal hypertrophy, tubulointerstitial fibrosis, and glomerulosclerosis by mitigating oxidative stress and inflammation in STZ‐induced diabetic mice.^125^ Dapagliflozin has also been shown to decrease OGT expression and prevent high glucose‐induced O‐GlcNAcylation, inhibiting RIF and tubular injury in STZ‐treated diabetic mice (Figure 5b).^126^ Moreover, ipragliflozin, another SGLT‐2i inhibits O‐GlcNAcylation of megalin and restores its endocytic function, and ameliorates proximal tubule protein overload, abnormal mitochondrial morphology, renal oxidative stress, and tubulointerstitial fibrosis in a non‐obese diabetic model of hypoinsulinemia (Figure 5b).^31^ These studies support that SGLT‐2i may attenuate HBP flux and inhibit O‐GlcNAcylation by blocking glucose entry into proximal tubule cells. However, the beneficial effects of SGLT‐2i attributed to inhibition of HBP flux and O‐GlcNAcylation in the kidney needs further investigations.

### 4.3 RAAS inhibitors

Angiotensin II contributes to the progression of diabetic kidney injury.^127^, ^128^, ^129^, ^130^ Hyperglycemia increases angiotensinogen gene expression in mesangial cells, glomerular endothelial cells, and PTECs.^131^, ^132^, ^133^ In PTECs, high glucose induces angiotensin gene expression, proximal tubular cell hypertrophy, and interstitial fibrosis through activation of HBP.^87^ RAAS inhibitors prevent hyperglycemia‐induced OGT expression and O‐GlcNAcylation, which is accompanied by an increase in OGA in PTECs (Figure 5c).^15^ Moreover, RAAS inhibitors prevent the elevation of O‐GlcNAcylation and ameliorate diabetes‐induced kidney damage via inhibition of Akt/eNOS phosphorylation and HSP72 expression in STZ‐induced diabetic rats (Figure 5c).^15^ Taken together, RAAS inhibitors may represent as new therapeutic targets for the treatment of diabetic nephropathy through their effects on O‐GlcNAcylation.

### 4.4 Lipoic acid

There is substantial evidence that increased oxidative stress may be involved in the pathogenesis of diabetic nephropathy.^134^, ^135^ Lipoic acid (LA), with its dual antioxidant and glucose‐lowering properties, may play an important role in the prevention of kidney injury and other complications of diabetes. Recently, LA has been shown to reduce oxidative stress markers in the renal cortex of STZ‐induced diabetic rats.^136^ In addition, it was found that renal tissues of diabetic rats treated with LA showed an increased expression of CuZn‐superoxide dismutase (SOD) and catalase with a decrease in the level of O‐GlcNAcylation of HSP70 and HSP90, and a decrease in cellular O‐GlcNAcylation of extracellular regulated kinases and p38 (Figure 5d).^137^ Moreover, LA decreases O‐GlcNAcylation of CuZn‐SOD and increases CuZn‐SOD gene expression and its enzymatic activity in STZ‐induced diabetic rats.^138^ These observations suggest that LA administration actions a renal protective response against diabetes‐induced oxidative injury in kidney tissue through an O‐GlcNAcylation‐dependent mechanism.

## 5 CONCLUSIONS AND FUTURE DIRECTIONS

**FIGURE 6:**
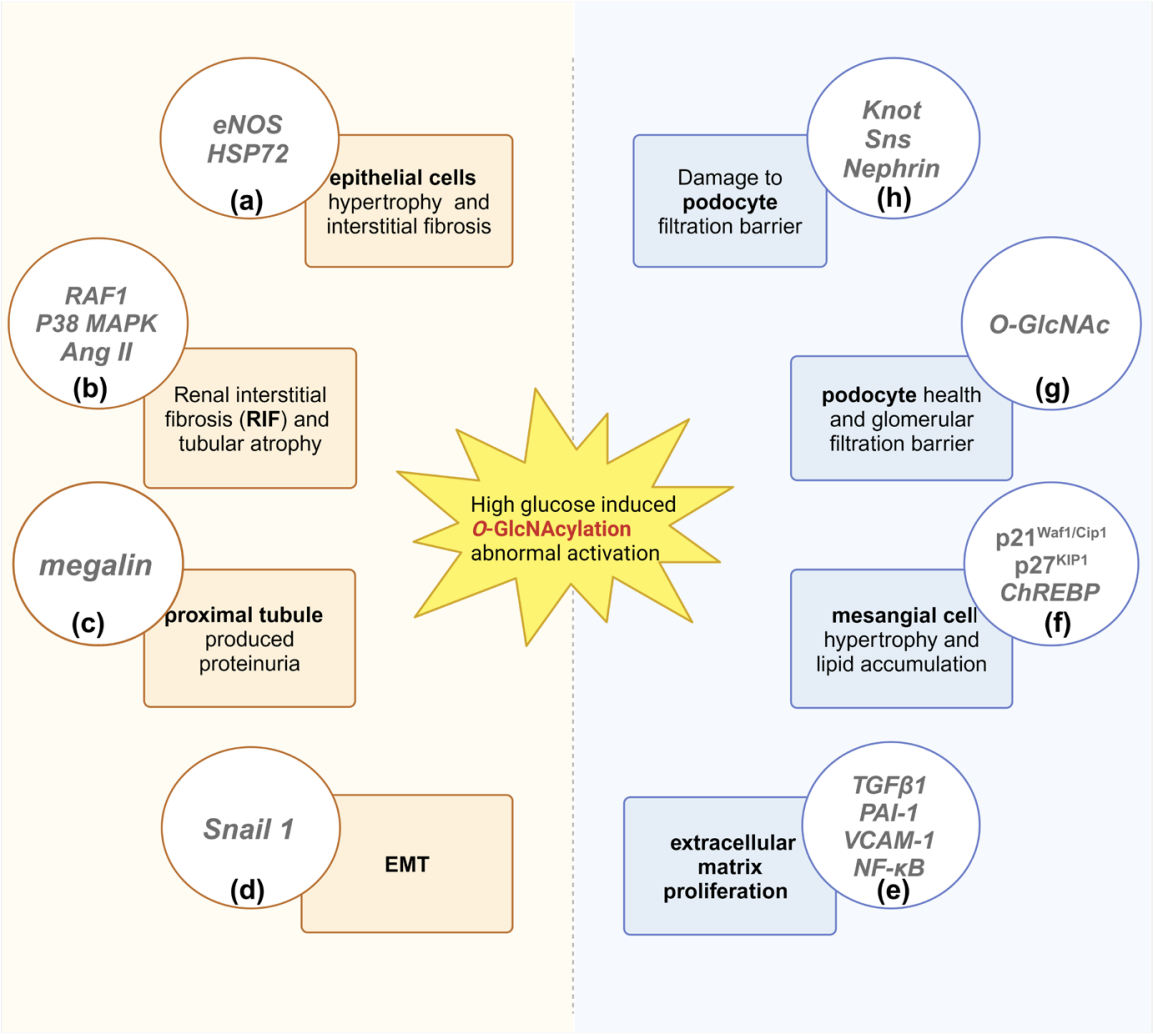
The relationship between abnormal activation of O‐GlcNAcylation and diabetic nephropathy. (a) In renal tubular epithelial cells (PTEC), O‐GlcNAc levels increase and the expressions of eNOS and HSP72 decrease, promoting renal fibrosis and accelerating the progression of CKD. (b) O‐GlcNAcNA acylation increases, inducing the expression of TGF‐β1 and Ang II, causing renal interstitial fibrosis (RIF), and destroying the structure and function of renal tubules. (c) O‐GlcNAc levels increase and megalin is downregulated in PTEC, resulting in proteinuria. (d) High glucose leads to an increase in the expression of O‐GlcNAcylation and an increase in the stability of Snail1, leading to EMT. (e) TGF β1, PAI‐1, VCAM‐1, and NF‐κB promote the development of CKD by inhibiting the degradation of extracellular matrix, increasing matrix protein accumulation, promoting the infiltration of inflammatory cells into the glomerulus. (f) Increased O‐GlcNAcylation levels induce the expression of p21^Waf1/Cip1^ and p27^KIP1^ in mesangial cells, arrest the cell cycle, inhibit cell growth and induce hypertrophy. ChREBP is involved in lipid accumulation in mesangial cells and renal fibrosis, leading to further deterioration of the kidney. (g) A certain level of O‐GlcNAcylation is essential for podocyte maturation and maintenance of normal structure and function. (h) Highly expressed O‐GlcNAcylation will activate Knot, reduce the expression of downstream Nephrin/Sns, destroy the integrity of the podocyte filtration barrier, and affect glomerular filtration function.

Aberrant activation of O‐GlcNAc modification contributes to the onset and progression of diabetic nephropathy (Figure 6). O‐GlcNAcylation of proteins occur in renal mesangial cells, mediating lipid deposition, inhibiting cell growth, inducing hypertrophy, increasing production of extracellular matrix proteins, and causing mesangial cell apoptosis. Moreover, O‐GlcNAc modification is essential for maintaining podocyte health and regulates glomerular filtration. In addition, O‐GlcNAc modification destroys PTEC structure and function and contributes to EMT during RIF. O‐GlcNAc modification activates proinflammatory responses, inhibits the degradation of extracellular matrix, and promotes RIF, leading to proteinuria and kidney damage and promoting the development of diabetic nephropathy. Drugs with nephroprotective properties (e.g. SGLT‐2i and RAAS inhibitors) have been shown to inhibit O‐GlcNAcylation. Although the significance of inhibition of O‐GlcNAcylation is still not fully understood, it may provide a novel target for the treatment of diabetic nephropathy.

## AUTHOR CONTRIBUTIONS

All authors have contributed to the discussion and manuscript writing and approved the submitted manuscript.

## FUNDING INFORMATION

This work was supported by Natural Science Foundation of Jilin Province (YDZJ202301ZYTS175 to BXQ), Jilin Province Outstanding Youth Fund Project (20240101007JJ to XDL), Diabetes UK (21/0006329 and 22/0006477 to LK), and British Heart Foundation (PG/18/56/33935 to LK).

## CONFLICT OF INTEREST STATEMENT

The authors declare no conflict of interests.

## Data Availability

The data that support the findings of this study are available from the corresponding author upon reasonable request.
